# Histology and fossil diagenesis of a pterosaur tooth from the Crato Formation (Lower Cretaceous of Brazil)

**DOI:** 10.1002/ar.70093

**Published:** 2025-11-11

**Authors:** Tito Aureliano, Matheus P. S. Rocha, Francisco P. Lima‐Filho, Aline M. Ghilardi

**Affiliations:** ^1^ Department of Biological Chemistry Regional University of Cariri (URCA) Crato Brazil; ^2^ Diversity, Ichnology and Osteohistology Research Group (DINOlab) Federal University of Rio Grande do Norte (URFN) Natal Brazil; ^3^ AKCIT‐IMD Federal University of Rio Grande do Norte (UFRN) Natal Brazil; ^4^ Museu Câmara Cascudo, Universidade Federal do Rio Grande do Norte (UFRN) Natal Brazil; ^5^ Laboratório de Estudos Estratigráficos (LAE), Department of Geology Federal University of Rio Grande do Norte (URFN) Natal Brazil

**Keywords:** Archosauria, morphology, paleontology, petrography, sedimentology

## Abstract

Pterosaur dental biology remains poorly understood despite its importance for comprehending feeding strategies and flight adaptations. Here, we present the first comprehensive histological analysis of an ornithocheiriform pterosaur tooth from the Lower Cretaceous Crato Formation (Santana Group, Northeast Brazil). Specimen LPP‐UFRN 3002 exhibits thin prismless enamel (61 μm thickness), orthodentine with dense tubule networks, and 82 von Ebner lines indicating rapid tooth formation. Quantitative comparison across major clades of extinct reptiles reveals that pterosaurs demonstrate the most accelerated tooth development among archosauriforms, with formation times of 54–82 lines compared to 185–933 lines in non‐avialan dinosaurs, 50–283 lines in crocodyliforms, and 342–426 lines in mosasaurs. The rapid tooth development and thin enamel documented in ornithocheiriform pterosaurs could enable the maintenance of lightweight skulls, essential for powered flight, while ensuring continuous replacement for effective prey capture. The specimen preserves microstructural detail despite diagenetic calcite permineralization and partial focalized substitution, a characteristic of the Crato Formation carbonate environment. These findings demonstrate that ornithocheiriform pterosaurs evolved a unique dental strategy, prioritizing replacement efficiency over individual tooth durability, which contrasts with the extended formation times characteristic of large‐bodied mosasaurs and herbivorous dinosaurs. This research establishes dental histology as critical for understanding pterosaur paleobiology and provides quantitative data for reconstructing ecosystem dynamics throughout the Mesozoic.

## Graphical abstract

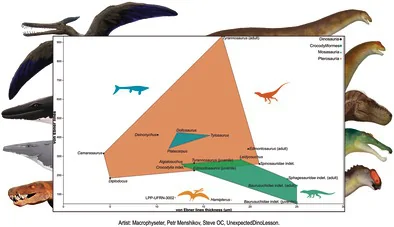

## 1 INTRODUCTION

Pterosaurs represent one of the most remarkable evolutionary innovations in vertebrate history, achieving powered flight through dramatic anatomical reorganization (Jagielska & Brusatte, 2021). They also developed sophisticated dental adaptations that supported diverse feeding strategies throughout their 150‐million‐year evolutionary history (Bestwick et al., 2018, 2020; Cerda & Codorniú, 2023; Pêgas et al., 2025). Their dental morphology evolved from the simple conical teeth of the non‐pterodactyloids to the specialized filter‐feeding apparatus of ctenochasmatids and the robust crushing dentitions of dsungaripterids, reflecting ecological diversification across Mesozoic ecosystems (Bestwick et al., 2020; Cerda & Codorniú, 2023; Ősi, 2011; Pêgas et al., 2025; Zhou & Fan, 2025). Despite extensive morphological studies documenting this dental diversity, the fundamental aspects of pterosaur tooth biology remain critically understudied. Recent advances in dental microwear texture analysis have revealed that pterosaurs occupied diverse dietary niches, from piscivory in *Rhamphorhynchus* to generalist invertebrate consumption in Pterodactylus and vertebrate predation in *Dimorphodon* (Bestwick et al., 2020). However, these functional insights stand in contrast to the limited understanding of the underlying developmental mechanisms that produced this dental diversity (Bestwick et al., 2020; Chen et al., 2023; Ciaffi & Bellardini, 2023, 2024; Zhou & Fan, 2025).

Knowledge gaps in pterosaur dental histology significantly limit our understanding of feeding ecology, metabolic constraints, and evolutionary responses to Mesozoic environmental changes (Bestwick et al., 2018, 2020; Cerda & Codorniú, 2023; Chen et al., 2023; Pêgas et al., 2025). While morphological analyses have established that pterosaur teeth varied substantially in shape, size, and arrangement, the developmental rates, replacement patterns, and histological organization underlying this diversity remain poorly characterized (Cerda & Codorniú, 2023; Chen et al., 2023; Ciaffi & Bellardini, 2023, 2024; Elias et al., 2007; Ősi, 2011; Pêgas et al., 2025; Zhou & Fan, 2025). Recent histological investigations have begun to illuminate pterosaur dental microstructure, revealing thin enamel layers and rapid development times in taxa such as *Hamipterus*, which exhibits 70 von Ebner incremental growth lines and thin prismless enamel (Chen et al., 2023). Similarly, the filter‐feeding pterodactyloid pterodaustrinins, including *Pterodaustro*, show unusual periodontal structures and distinctive tooth implantation patterns that differ fundamentally from other pterosaurs (Cerda & Codorniú, 2023; Pêgas et al., 2025). In *Pterodaustro*, the tooth implantation is characterized by an ankylothecodont attachment, a direct fusion of the tooth base with the alveolar bone, lacking the typical periodontal ligament observed in other pterosaurs. The periodontium shows a reduced or absent periodontal space, replaced by cellular cementum directly joining tooth and bone, as well as a shallow alveolar socket (Cerda & Codorniú, 2023). However, comprehensive comparative analyses across pterosaur clades and with other major reptilian groups remain lacking, preventing assessment of whether rapid tooth formation represents a consistent pterosaurian adaptation or varies with feeding ecology and phylogenetic position (Chen et al., 2023; Ciaffi & Bellardini, 2023, 2024).

The evolutionary context of pterosaur dental biology becomes particularly significant when considered alongside the dental strategies of contemporary dinosaurs, crocodyliforms, and marine reptiles, as tooth development patterns directly reflect physiological constraints, ecological roles, and competitive pressures within Mesozoic food webs (Bestwick et al., 2018, 2020). Tooth histology in extinct reptiles reveals fundamental differences in developmental strategies: non‐avian dinosaurs typically exhibit extended tooth formation times ranging from 185 to 933 von Ebner lines, with herbivorous taxa investing heavily in wear‐resistant enamel optimized for extended functional lifespans (D'Emic et al., 2013, 2019, 2025; Erickson, 1996; Heckeberg & Rauhut, 2020). Crocodyliforms display intermediate formation patterns (50–283 lines) with notably thick daily dentin increments, while mosasaurs consistently show prolonged development periods (342–426 lines) comparable to large dinosaurs (Chinsamy et al., 2012; Gren & Lindgren, 2014; Ricart et al., 2021). These divergent patterns raise critical questions about pterosaur dental evolution: Did the biomechanical demands of flight impose unique constraints on tooth development? How do pterosaur formation rates compare quantitatively to contemporary reptilian clades? Understanding these comparative patterns contributes to broader questions regarding the evolution of dental complexity in fossil reptiles and provides insights into the relationship between aerial locomotion and feeding apparatus design (Cerda & Codorniú, 2023; Chen et al., 2023; Chinsamy et al., 2012; D'Emic et al., 2013, 2019, 2025; Erickson, 1996; Gren & Lindgren, 2014; Ősi, 2011; Pêgas et al., 2025).

The exceptional preservation conditions of certain Lagerstätten, particularly the Lower Cretaceous deposits of the Santana Group in Northeast Brazil, provide unprecedented opportunities to examine pterosaur dental histology with remarkable fidelity (Cabral et al., 2019; Fambrini et al., 2020; Neumann & Cabrera, 1999). The Crato Formation within this group represents a classic Konservat‐Lagerstätte, where anoxic bottom waters, hypersaline conditions, and rapid burial in fine carbonate sediments created conditions for exceptional fossil preservation (Cabral et al., 2019; Heimhofer et al., 2010). However, diagenetic processes in carbonate‐dominated lacustrine environments affect mineralized tissues differently than soft tissues, with calcite permineralization and recrystallization potentially obscuring or destroying delicate histological features while simultaneously stabilizing specimens through early cementation (Araújo et al., 2020; Cabral et al., 2019; Heimhofer et al., 2010). Understanding these diagenetic pathways is essential for interpreting histological data from Crato Formation specimens and for assessing the preservational limits of tooth microstructures in similar depositional environments. The taphonomic context, therefore, becomes integral to evaluating what histological information can reliably be extracted from fossil teeth and how diagenetic alterations may bias our understanding of original tissue organization.

Here, we describe the histology and diagenesis of an ornithocheiriform pterosaur tooth (specimen LPP‐UFRN 3002) from the Crato Formation (Santana Group, Northeast Brazil) using integrated histological, petrographic, and comparative approaches. We provide a quantitative comparative analysis of von Ebner incremental line counts and thickness across 22 taxa representing pterosaurs, dinosaurs, crocodyliforms, and mosasaurs to assess developmental patterns and evolutionary strategies in tooth formation among major extinct reptilian clades (Chen et al., 2023; Chinsamy et al., 2012; D'Emic et al., 2013; Erickson, 1996; Gren & Lindgren, 2014; Heckeberg & Rauhut, 2020; Ricart et al., 2021). We quantify enamel thickness, dentin organization, and growth increment spacing to establish tooth formation time and daily dentin deposition rates, enabling direct comparison with published data from other archosaurs and marine reptiles. Additionally, we characterize diagenetic processes affecting tooth preservation in the Crato Formation carbonate environment, including calcite permineralization patterns and recrystallization impacts on histological features, contributing to understanding taphonomic pathways in this Konservat‐Lagerstätte. Our results contribute to reconstructing pterosaur dental function and its evolutionary significance within the broader context of reptile radiation and diversification during the Mesozoic Era.

## 2 MATERIALS AND METHODS

### 2.1 Institutional abbreviations

IVPP, Institute of Vertebrate Paleontology and Paleoanthropology, Chinese Academy of Sciences, Beijing, China; LPP‐UFRN, Laboratório de Paleontologia de Vertebrados, Universidade Federal do Rio Grande do Norte, Natal, Brazil; MCF‐PVPH, Museo “Carlos Ameghino”‐Paleontología de Vertebrados, Provincia del Neuquén, Argentina.

### 2.2 Specimen, locality and horizon

Specimen LPP‐UFRN 3002 consists of an isolated ornithocheiriform pterosaur tooth crown preserved in fine‐grained limestone from the Aptian Crato Formation, Araripe Basin (Fambrini et al., 2020; Neumann & Cabrera, 1999). The specimen represents a nearly complete crown lacking the root and any associated alveolar bone or cementum from the root region. The crown shows excellent preservation with no evidence of erosion or deformation. The specimen was collected by F.P.L.F. in 2019 during field classes in a quarry near Nova Olinda municipality, southern Ceará state, Northeast Brazil.

### 2.3 Tooth morphology

The general dental morphological description follows the terminology proposed by Hendrickx et al. (2015) for theropod dinosaur specimens. Considering that the dental morphology of ornithocheiriforms does not strictly follow dinosaur dentition, certain terms follow the usage patterns of Ciaffi and Bellardini (2024), with the term “carina” referring to the apicobasal constriction of the mesial and distal surfaces of the crown, which does not correspond exactly to the clear, well‐defined crest described by Hendrickx et al. (2015). The specimen was observed and photographed with an iPhone 13 Pro.

### 2.4 Paleohistology and petrography

We sampled the tooth still embedded in the limestone. The tooth was sectioned in the coronal plane (dividing the tooth into labial and lingual portions) along the mesiodistal axis at the exact middle of the tooth. We applied PaleoBOND® penetrant stabilizer to improve sample resistance. It was embedded in Araldite 2020® epoxy resin. The sample was later ground to a thickness of approximately 60 μm. For general thin‐section maps, we used a Meyer Instruments PathScan Enabler. For detailed photomicrographs, we used an Olympus BX53M petrographic microscope equipped with both parallel and crossed nicols, with the lambda compensator. We followed Chen et al. (2023) for tooth histology description. We followed the classification of Embry and Klovan (1971) and Terra et al. (2010) for carbonate rock petrography.

### 2.5 Von Ebner line quantification and comparative analysis

We quantified von Ebner incremental growth lines following the methodology of Erickson (1996). Lines were counted in well‐preserved dentin regions under crossed polarized light using an Olympus BX53M petrographic microscope. Von Ebner line thickness was measured perpendicular to line orientation using ImageJ software version 1.53 (Schneider et al., 2012), calibrated against the microscope scale bar, with measurements taken at regular intervals along the dentin. We performed quantitative comparative analysis involving 22 taxa, including 8 dinosaurs (2 ornithischians, 4 theropods, and 2 sauropods), 3 pterosaurs (including LPP‐UFRN 3002), 7 crocodyliforms, and 4 mosasaurs (Chen et al., 2023; Chinsamy et al., 2012; Ciaffi & Bellardini, 2023; D'Emic et al., 2013; Erickson, 1996; Gren & Lindgren, 2014; Heckeberg & Rauhut, 2020; Ricart et al., 2021). Comparative data were compiled from published literature (see Table S1) and analyzed to assess patterns of tooth formation time and daily dentin deposition rates across major extinct reptilian clades.

## 3 RESULTS

### 3.1 Systematic paleontology and tooth morphology

Order Pterosauria Kaup, 1834.

Suborder Pterodactyloidea Plieninger, 1901.

Lanceodontia Andres et al., 2014.

Ornithocheiriformes Andres, 2021.

**FIGURE 1:**
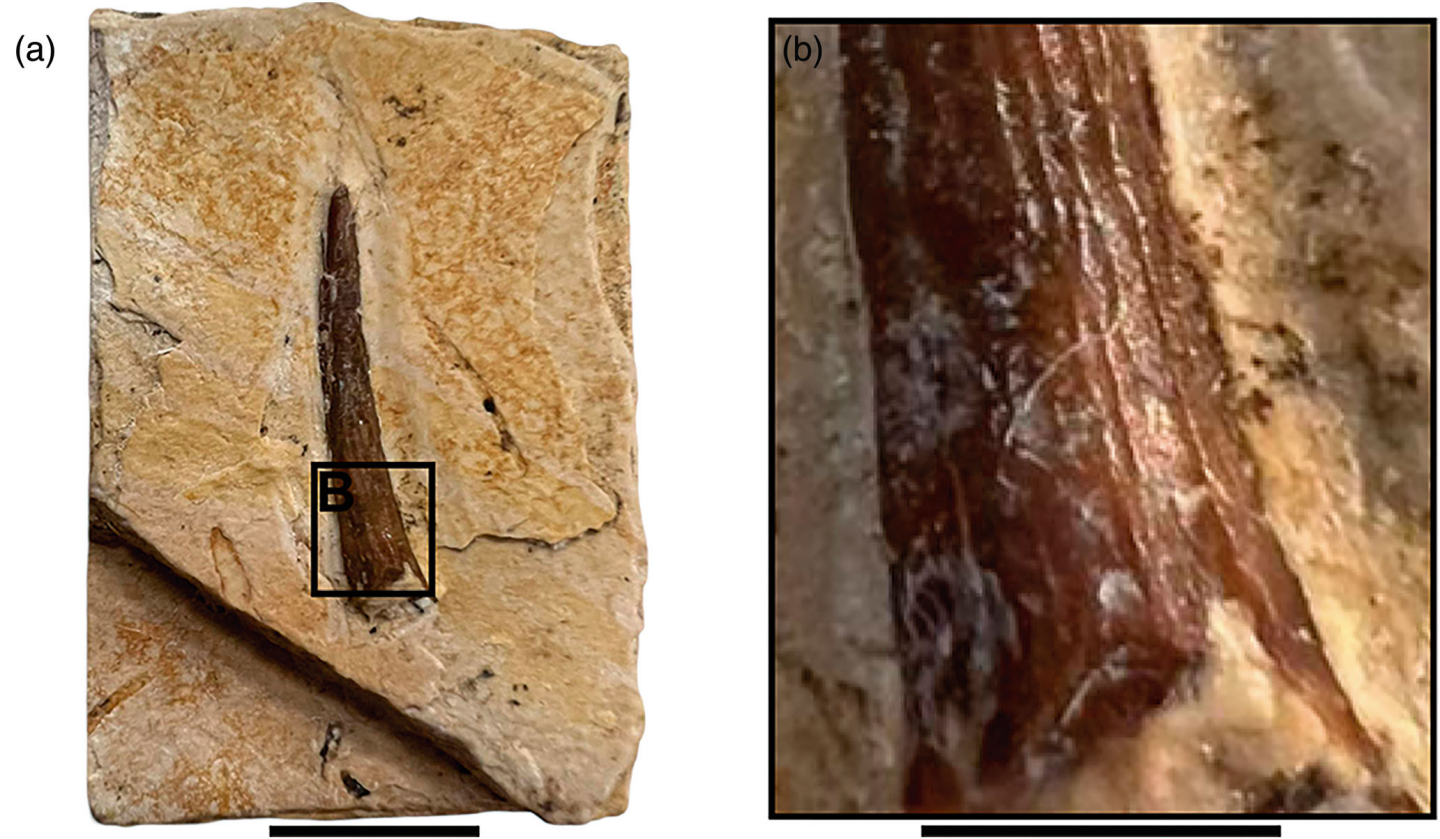
Isolated ornithocheiriform pterosaur tooth LPP‐UFRN 3002 embedded in limestone from the Lower Cretaceous Crato Formation, Ceará, Brazil (a), and detail on the ornamentation (b). Scale bar in a = 10 mm; in b = 30 mm.

Conservation Status and General Morphology: The specimen is preserved in a small block of fine‐grained limestone. The tooth represents an almost complete, isolated dental crown, preserving only the crown itself. No root and alveolar bone have been preserved. The crown exhibits no signs of erosion or deformation, including twisting, shearing, or bending. The isolated crown is apicobasally elongated, slender, and conical (conidont) in shape, with gentle recurvature and a circular cross‐section at the base. This morphology is consistent with anterior tooth positions in ornithocheiriform pterosaurs, as evidenced by isolated specimens such as RH45 (assigned to Morphotype 1 by Ciaffi & Bellardini, 2024) and articulated skulls of *Anhanguera* (Kellner & Tomida, 2000) and *Ludodactylus* (Frey et al., 2003), where anterior teeth exhibit similar elongated, conical morphology with gentle recurvature. It differs markedly from the robust, laterally compressed teeth of theropod dinosaurs or the bulbous, multi‐cusped teeth seen in crocodyliforms (Aureliano et al., 2015; Chen et al., 2023; Ciaffi & Bellardini, 2024; Delcourt et al., 2024; Hendrickx et al., 2015; Martill et al., 2018; Ricart et al., 2021). The enamel surface exhibits distinct ornamentation, consisting of fine anastomosed canals and a wrinkled appearance, paralleling the vertically directed, crenulated enamel ridges (Figure 1b) observed in ornithocheiriform pterosaur teeth from the Aptian–Albian of Tunisia, Brazil, and Argentina (Ciaffi & Bellardini, 2024; Martill et al., 2018). Such ornamentation is typical of anhanguerids (e.g., *Coloborhynchus*, *Ludodactylus*) and contrasts with the smooth or grooved enamel surfaces of most non‐pterosaurian archosaurs (Aureliano et al., 2015; Ciaffi & Bellardini, 2024; Delcourt et al., 2024; Hendrickx et al., 2015; Martill et al., 2018; Ricart et al., 2021; Rocha et al., 2025). The specimen is here referred to Pterosauria based on a combination of morphological and histological features characteristic of this clade, following comparative criteria as outlined in Martill et al. (2018).

### 3.2 Petrography and taphonomy

**FIGURE 2:**
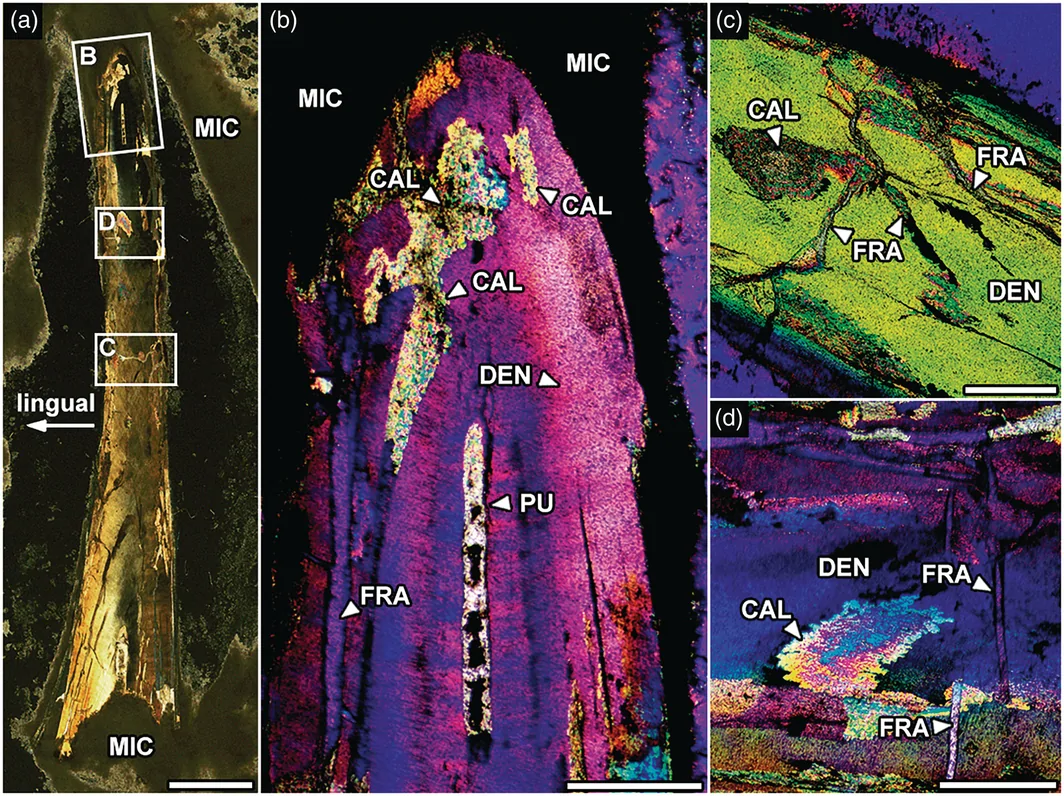
Petrography of LPP‐UFRN 3002 from the Lower Cretaceous (Aptian) Crato Formation, Araripe Basin (Ceará state, Brazil). (a) Bioclast embedded in the mudstone. (b) Detail of the tip of the crown showing diagenetic calcite permineralization and recrystallization. (c, d) Detail of the tooth crown, showing diagenetic fractures and calcite recrystallization on the dentin. Polarizing light under crossed nicols (a–d) and lambda compensator in b–d. Rotation of 45° in C and 90° in D (both clockwise). CAL, diagenetic calcite; DEN, dentin; FRA, diagenetic fracture; MIC, carbonatic micrite; PU, pulp. Scale bar in a = 300 μm; b‐d = 500 μm.

**FIGURE 3:**
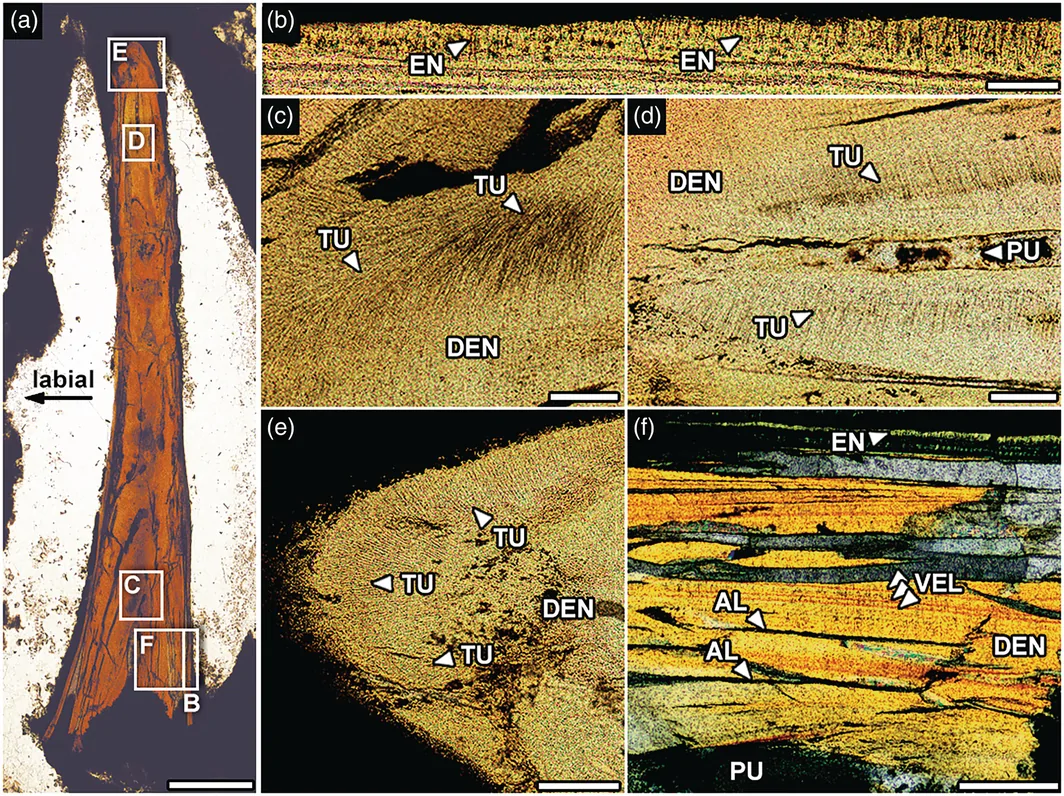
Histology of the ornithocheiriform tooth LPP‐UFRN 3002 from the Lower Cretaceous (Aptian) Crato Formation, Araripe Basin (Ceará state, Brazil). (a) Tooth longitudinal section. (b) detail of the prismless enamel. (c) radiating tubules in the orthodentine near the apical zone. (d) detail of the tubules near the pulp. (e) radiating tubules with varying angles at the tip of the tooth. (f) base of the crown showing enamel, orthodentine, and pulp. Note the Andresen and von Ebner lines. Polarizing light under parallel (a–e) and crossed (f) nicols. Rotation of 90° in b, d–f, and 120° in C (all clockwise). CAL, diagenetic calcite; DEN, dentin; FRA, diagenetic fracture; MIC, carbonatic micrite; PU, pulp. Scale bar in a = 300 μm; b = 100 μm; c–e = 200 μm; f = 500 μm.

The sample is matrix‐supported and consists of carbonaceous micrite (Figures 2 and 3). Hence, this setup is compatible with the definition of a mudstone (Embry & Klovan, 1971; Terra et al., 2010). The single major bioclast present in the sample is the pterosaur tooth (Figure 2a). This element is fractured at the base, and the root has not been preserved. There are longitudinal fractures following dentin growth lines (Figure 2b,c). There are also orthogonal fractures at the upper portion of the tooth (Figure 2c,d). There is permineralized calcite following those fractures, and there are also areas of dentin partially destroyed due to calcite recrystallization (Figure 2b–d).

### 3.3 Paleohistology

There is a thin outer layer of simple, prismless enamel covering the tooth crown (Figure 3b). The enamel presents a very low absolute thickness (61 μm; relative thickness to crown radius = 0.04), and tubules are present (Figure 3b). The surface shows limited wear at the microscopic scale. We detected enamel around the entire crown surface, but failed to detect it only at the very tip of the tooth. The dentin (orthodentine) contains dense networks of tubules (~20 per 100 μm) with a greater concentration radiating out of the pulp cavity near the basal zone and the tip (Figure 3c–e). The coronal pulp is present as a slender, spindle‐shaped central cavity tunnel (Figure 3a,d,f), with a diameter varying from 425 μm (at the basal zone) to 100 μm (at the apical zone).

### 3.4 Tooth growth rate estimation in LPP‐UFRN 3002

Two types of incremental lines were detected in the dentin (Figure 3f): Andresen and von Ebner lines. Anderssen lines form in response to periodic variations in collagen fiber orientation, and were here overprinted by the thick, dark, diagenetic minerals. The short‐period von Ebner lines deposit between the Andresen lines and present a clear and subtle appearance under polarizing light. The lines of von Ebner have been suggested as indicators of daily increments of dentin mineralization (Chen et al., 2023; Ricart et al., 2021), and were observed at relatively varying distances to each other (Figure 3f). The number of von Ebner lines between Andresen lines ranges from 5 to 19, and the widths vary from 7 to 19 μm (mean = 12 μm). A total of 82 von Ebner lines were counted.

### 3.5 Tooth growth rates in fossil reptiles

**FIGURE 4:**
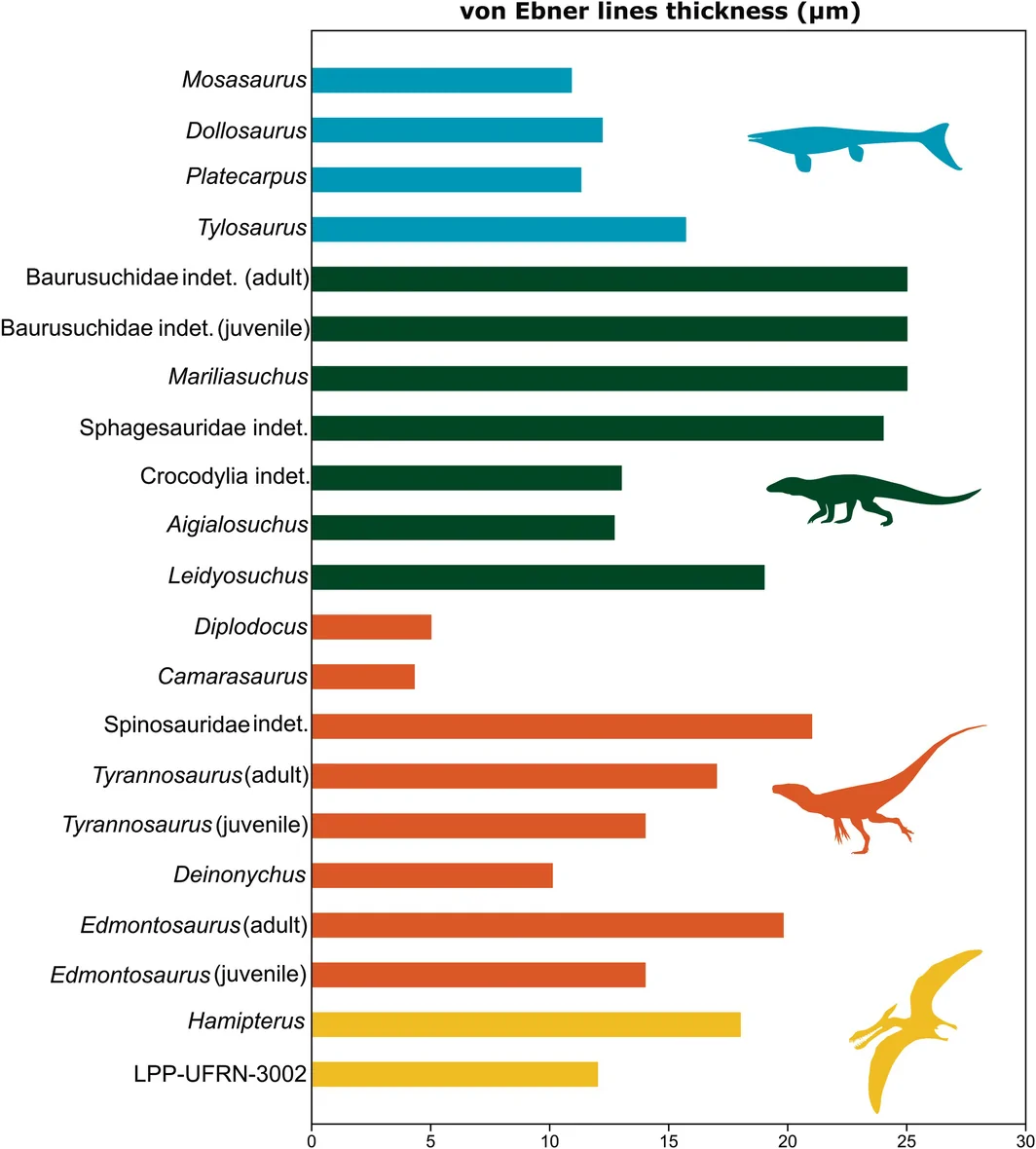
Quantification of dental growth marks in fossil reptiles. Average values for von Ebner line thickness of pterosaurs, dinosaurs, crocodyliforms, and mosasaurs. See Table S1 for specimen numbers and references. Silhouettes by Phylopic (https://creativecommons.org/licenses/by‐sa/3.0/), courtesy of Matthew Crook, T. Michael Keesey, Tasman Dixon and thefunkmonk (adapted).

**FIGURE 5:**
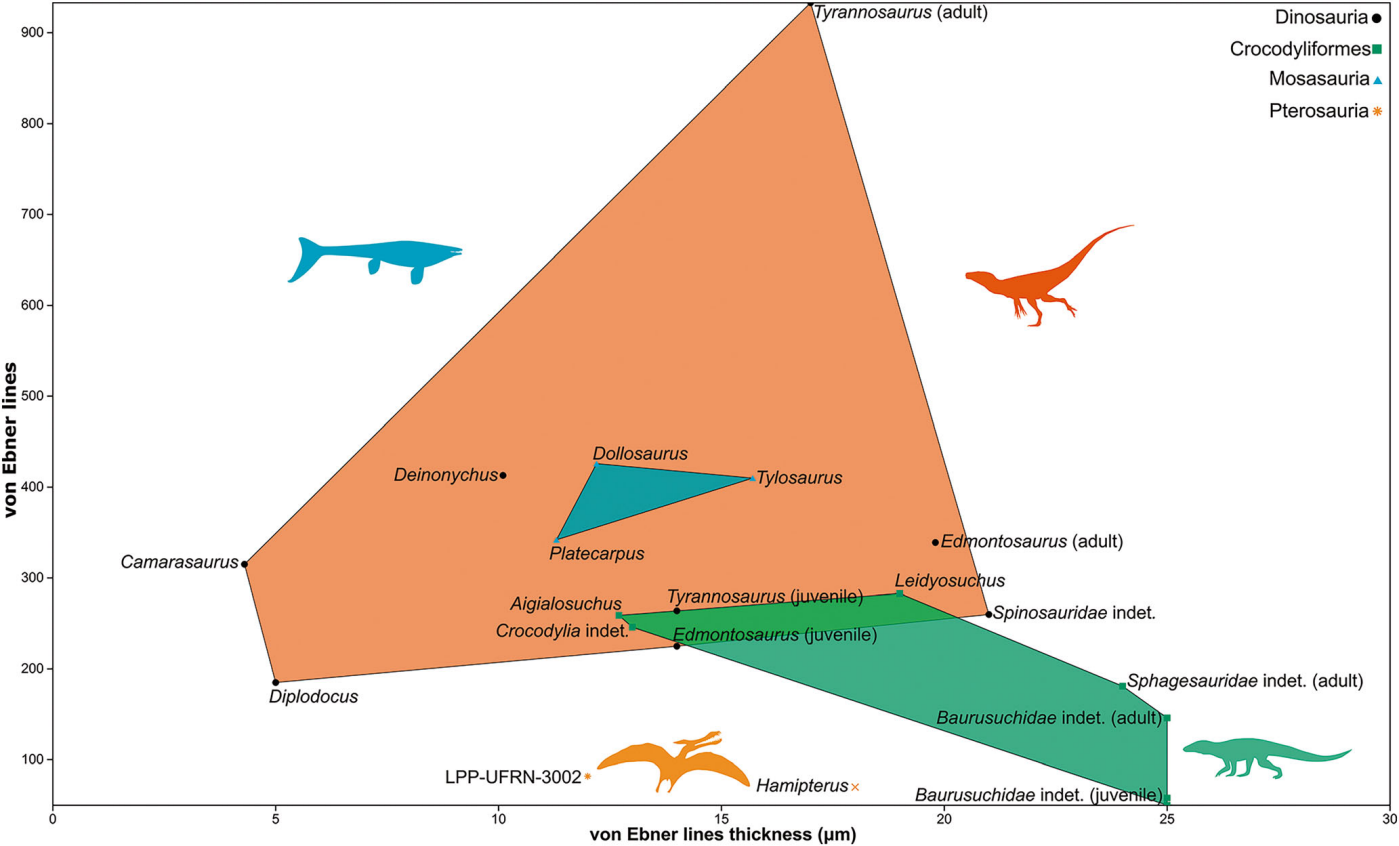
Bivariate plot displaying the number of von Ebner lines against von Ebner line thickness, to summarize tooth growth strategies in pterosaurs, dinosaurs, crocodyliforms, and mosasaurs. Silhouettes by Phylopic (https://creativecommons.org/licenses/by‐sa/3.0/), courtesy of Matthew Crook, T. Michael Keesey, Tasman Dixon, and thefunkmonk (adapted).

The analysis of von Ebner lines across pterosaurs, dinosaurs, crocodyliforms, and mosasaurs reveals distinct patterns in tooth growth strategies among the four major clades examined (Table S1 and Figures 4 and 5). Pterosaurs demonstrate the lowest von Ebner line counts, ranging from 54 to 82 lines (mean = 68.7), with thickness values of 12–18 μm (mean = 15.0 μm) where available (Chen et al., 2023; Ciaffi & Bellardini, 2023). Dinosaurs exhibit significantly higher von Ebner line counts, spanning 185 to 933 lines (mean = 366.8), representing the greatest range among all groups (D'Emic et al., 2013; Erickson, 1996), while thickness measurements range from 10.1 to 21.0 μm (mean = 16.0 μm). Crocodyliforms show intermediate von Ebner line counts of 50 to 283 lines (mean = 174.7) but display the greatest thickness values (Ricart et al., 2021), ranging from 12.7 to 25.0 μm (mean = 20.5 μm). Mosasaurs consistently exhibit high von Ebner line counts, similar to those of dinosaurs (Chinsamy et al., 2012; Gren & Lindgren, 2014), ranging from 342 to 426 lines (mean = 392.7), but with more conservative thickness values of 10.9 to 15.7 μm (mean = 12.5 μm).

## 4 DISCUSSION

### 4.1 Remarks on pterosaur tooth histology

The histological analysis of the ornithocheiriform LPP‐UFRN 3002 contributes to the understanding of pterosaur tooth development and the dental evolution within this clade. The 82 von Ebner lines counted in LPP‐UFRN 3002 align with the pattern observed in *Hamipterus* IVPP V 32250.5 (Chen et al., 2023), 2023), which has 70 von Ebner lines, and with the ornithocheiriform specimen MCF‐PVPH‐879‐2 from Argentina (Ciaffi & Bellardini, 2023), which exhibits 54 von Ebner lines. The consistency of these values (54–82 lines) across different ornithocheiriform taxa from geographically and temporally distinct formations suggests that rapid tooth formation was a stable evolutionary trait within this group. The von Ebner line thickness in our specimen (mean = 12 μm) is consistent with values reported for *Hamipterus* (18 μm; Chen et al., 2023). This thickness falls within the range typically observed in archosaur teeth (Chen et al., 2023; Chinsamy et al., 2012; D'Emic et al., 2013; Erickson, 1996; Gren & Lindgren, 2014; Heckeberg & Rauhut, 2020; Ricart et al., 2021) and suggests comparable physiological processes of daily dentin mineralization across pterosaur taxa.

The enamel of LPP‐UFRN 3002 is particularly noteworthy when viewed in the broader context of pterosaur dental evolution. Our specimen shows a thin outer layer of simple, prismless enamel with very low absolute thickness (61 μm; relative thickness to crown radius = 0.04). This limited enamel coverage and thickness are consistent with the general pterosaur dental pattern and reflect the emphasis on rapid tooth replacement rather than enhanced tooth durability (Chen et al., 2023; Chinsamy et al., 2012). The fragility of pterosaur enamel, as observed in our specimen and noted in *Hemipterus* (Chen et al., 2023), supports the interpretation that these flying reptiles relied heavily on frequent tooth turnover rather than robust individual teeth.

The dental microstructure of LPP‐UFRN 3002, with its orthodentine containing dense networks of tubules (~20 per 100 μm), represents a fairly typical pterosaur condition (Chen et al., 2023; Ciaffi & Bellardini, 2023; Vidovic, 2010). The presence of both Andresen and von Ebner lines in our specimen provides data for understanding pterosaur tooth growth patterns, as such detailed histological information remains rare. The rapid tooth formation times (54–82 days) documented across ornithocheiriform pterosaurs support the hypothesis that these flying reptiles required efficient tooth replacement strategies to maintain functional dentitions adapted for piscivorous feeding (Chen et al., 2023; Ciaffi & Bellardini, 2023). However, the tooth histology of ornithocheiriform pterosaurs contrasts markedly with that of the specialized filter‐feeding ctenochasmatid pterodaustrinins (Cerda & Codorniú, 2023; Pêgas et al., 2025). While LPP‐UFRN 3002 and *Hamipterus* exhibit well‐preserved von Ebner lines, pterodaustrinins (including *Pterodaustro*) completely lack these incremental growth lines (Cerda & Codorniú, 2023; Pêgas et al., 2025), preventing assessment of tooth development rates. The enamel structure also differs significantly between these groups: ornithocheiriforms possess thin but detectable prismless enamel layers (61 μm in LPP‐UFRN 3002; ~25 μm in *Hamipterus*), whereas pterodaustrinins, including *Pterodaustro*, show only a thin birefringent enamel band with no observable incremental lines (Cerda & Codorniú, 2023; Chen et al., 2023; Pêgas et al., 2025). These fundamental differences demonstrate that pterosaur dental evolution involved not just variation in tooth morphology and replacement rates, but also changes in developmental aspects underlying tooth function; however, shifts in attachment mechanisms are only documented in certain filter‐feeding lineages and are not supported for ornithocheiriforms by our data (Cerda & Codorniú, 2023; Chen et al., 2023; Ciaffi & Bellardini, 2024; Vidovic, 2010; Zhou & Fan, 2025).

### 4.2 Remarks on tooth microstructure in pterosaurs, dinosaurs, crocodyliforms, and mosasaurs

The comparative analysis of tooth formation rates across major archosaur clades and mosasaurs reveals fundamental differences in dental development strategies, with pterosaurs exhibiting unique characteristics that distinguish them from all other groups examined.

Pterosaurs demonstrated the most accelerated tooth development documented in our sample (Table S1 and Figure 4). The ornithocheiriforms LPP‐UFRN 3002, MCF‐PVPH‐879‐2, and *Hamipterus* IVPP V 32250.5 present 82, 54, and 70 von Ebner lines, respectively, suggesting dentin formation time in these taxa ranging from 60 to 80 days (Chen et al., 2023; Ciaffi & Bellardini, 2023). This contrasts markedly with dinosaur formation patterns, where *Edmontosaurus* exhibits 225–339 lines, *Deinonychus* shows 413 lines, and *Tyrannosaurus* requires 264–933 lines (Erickson, 1996). Sauropods display intermediate values, with *Camarasaurus* at 315 lines and *Diplodocus* at 185 lines (D'Emic et al., 2013). In the case of sauropods, D'Emic et al. (2025) noted variable replacement rates among clades, ranging from 2 to 3 months in the earliest macronarians and brachiosaurids, and 2–5 weeks in diplodocoids. Crocodyliforms exhibit formation times of 50–283 days (Ricart et al., 2021), spanning from rapid development in the notosuchian *Mariliasuchus* (50 lines) to extended formation in *Leidyosuchus* (283 lines). Mosasaurs consistently show extended formation periods comparable to large dinosaurs, ranging from 342 to 426 days (mean = 392.7 days) in taxa such as *Dollosaurus* and *Tylosaurus* (Gren & Lindgren, 2014).

Pterosaurs also exhibit moderate von Ebner line thickness (12–18 μm, mean = 15.0 μm), comparable to dinosaur values (10.1–21.0 μm, mean = 16.0 μm) but thinner than the robust lines characteristic of crocodyliforms (12.7–25.0 μm, mean = 20.5 μm). Mosasaurs display the most conservative thickness values (10.9–15.7 μm, mean = 12.5 μm), despite their extended formation times.

The enamel of LPP‐UFRN 3002 (61 μm thickness) exemplifies the pterosaur strategy of minimal enamel investment, representing among the thinnest layers documented in our sample of taxa. This prismless, simple enamel structure facilitates rapid development but produces mechanically fragile teeth requiring frequent replacement (Chen et al., 2023). Conversely, dinosaurs typically evolved thick, wear‐resistant enamel optimized for extended functional lifespans (D'Emic et al., 2013; Erickson, 1996; Heckeberg & Rauhut, 2020), while mosasaurs developed intermediate enamel thickness (approximately 190 μm in *Mosasaurus*; Chinsamy et al., 2012).

The rapid tooth development documented in pterosaurs could enable maintenance of lightweight skulls essential for powered flight while ensuring continuous replacement for effective prey capture (Chen et al., 2023). This strategy contrasts with the extended formation times characterizing large‐bodied aquatic predatory mosasaurs (Chinsamy et al., 2012; Gren & Lindgren, 2014) and herbivorous dinosaurs (D'Emic et al., 2013; Erickson, 1996), which evolved enhanced durability through increased formation times and robust enamel architecture. The pterosaur approach prioritized replacement efficiency over individual tooth longevity within the constraints of aerial locomotion (Chen et al., 2023).

### 4.3 Patterns of tooth development across extinct reptilian clades

The bivariate analysis of von Ebner line counts versus thickness (Figure 5) reveals distinct developmental strategies among major reptilian clades. Pterosaurs occupy a unique position characterized by low von Ebner counts (54–82 lines) and moderate thickness values (12–18 μm), clustering separately from all other groups. This pattern indicates accelerated tooth formation optimized for rapid replacement. Dinosaurs exhibit the broadest range, with herbivorous taxa such as *Edmontosaurus* and sauropods showing extended formation times (185–933 lines) that correlate with increased crown complexity and durability requirements. Crocodyliforms display the thickest daily increments (12.7–25.0 μm) combined with intermediate line counts, suggesting relatively rapid dentin deposition rates per day but variable total formation times depending on tooth size and ecology. Mosasaurs cluster with high line counts (342–426) but thin daily increments (10.9–15.7 μm), indicating prolonged, steady development of large, robust teeth suited for marine predation (Chinsamy et al., 2012; Gren & Lindgren, 2014). These divergent patterns reflect fundamental differences in dental ecology, with ornithocheiriform pterosaurs prioritizing lightweight, frequently replaced teeth for aerial piscivory (Chen et al., 2023), while most large‐bodied aquatic and terrestrial reptiles invested in tooth durability (Chinsamy et al., 2012; Currie et al., 1990; Delcourt et al., 2024; D'Emic et al., 2013, 2019, 2025; Erickson, 1996; Gren & Lindgren, 2014; Ricart et al., 2021).

### 4.4 Comments on the fossil diagenesis of the Crato formation

The diagenetic history of pterosaur tooth LPP‐UFRN 3002 shows the preservation mechanisms in the Crato Formation Konservat‐Lagerstätten. The tooth exhibits calcite permineralization along fractures, calcite recrystallization affecting dentin integrity, and longitudinal and orthogonal fracture development within carbonaceous micrite typical of this lacustrine deposit (Cabral et al., 2019; Heimhofer et al., 2010). These diagenetic modifications contrast with preservation documented for other fossil groups in the same unit, where anoxic bottom waters, hypersaline conditions, and rapid burial in fine sediments created conditions for soft‐tissue preservation (Cabral et al., 2019; Heimhofer et al., 2010; Osés et al., 2017). Insects from the Crato Formation show preservation through pyrite pseudomorphs replacing organic matter and phosphatization of tissues, maintaining three‐dimensional morphology through early lithification (Osés et al., 2016). Our pterosaur tooth experienced more extensive post‐depositional alteration. This differential preservation reflects distinct taphonomic pathways affecting mineralized versus organic tissues within the stratified lacustrine system. The diagenetic calcite in the tooth, including areas of dentin destruction due to recrystallization, suggests exposure to carbonate‐saturated pore fluids during burial (Araújo et al., 2020; Cabral et al., 2019; Heimhofer et al., 2010). This process facilitated the preservation of the fossil through early cementation while altering the microstructure of pre‐existing biomineral components in specific spots. This illustrates how selective the diagenetic environment of the Crato Formation is, where the same geochemical conditions that established this deposit as a Konservat‐Lagerstätte could also variably affect different organic materials, preserving soft tissues while altering vertebrate hard tissues with microscopic damage through calcite replacement and recrystallization processes.

### 4.5 Comments on the fossil teeth preservation across different geological contexts

The taphonomic patterns of fossilized teeth preservation become clearer when observing other geological and adaptive contexts (Beevor et al., 2021; Chen et al., 2023; Chinsamy et al., 2012; Cullen et al., 2023; Delcourt et al., 2024; Erickson, 1996; Gren & Lindgren, 2014; Hendrickx et al., 2019). The main reasons for enamel loss are morphological and/or biostratinomic, not diagenetic (Cullen et al., 2023; Delcourt et al., 2024). Tooth erosion may occur in consequence of adaptive feeding habits of certain organisms lacking lips (eg, crocodylians), while taxa with lips covering teeth protect and lubricate these organs (eg, mammals), improving their endurance (Cullen et al., 2023). Other adaptations also improve resistance, such as the iron‐coated teeth of *Varanus komodoensis* (LeBlanc et al., 2024). Most of the preservation and destruction factors observed in fossilized teeth are related to the paleoenvironmental sedimentological systems, and either to pre‐burial or post‐exhumation transport and weathering (Ciaffi & Bellardini, 2024; Delcourt et al., 2024). Among the diagenetic processes affecting preservation, lithostatic compression may deform cranial features and teeth, as possibly happened in *Chilesaurus* and *Nqwebasaurus*, increasing their procumbency (Choiniere et al., 2012; Hendrickx et al., 2019). However, biogenic micrite may partially obliterate visibility in teeth preserved inside calcareous concretions (Pêgas et al., 2025). Other diagenetic processes have been known to destroy dentin incremental lines (D'Emic et al., 2019), and the loss of the mesial carina (Ciaffi & Bellardini, 2024), but detailed descriptions are pending. Nonetheless, the most destructive diagenetic process remains when dentin is partially dissolved and recrystallized as calcite, as seen in the pterosaur LPP‐UFRN 3002, resulting in major information loss similar to other osteohistological contexts under similar chemical contexts (Aureliano et al., 2020).

## 5 CONCLUSIONS

The histological analysis of ornithocheiriform tooth LPP‐UFRN 3002 from the Crato Formation provides new data on pterosaur dental development and microstructure. Our specimen shows rapid tooth formation, completing dentin development in 82 von Ebner line counts. The tooth exhibits thin prismless enamel (61 μm thickness) and orthodentine with approximately 20 tubules per 100 μm, suggesting a structure optimized for replacement efficiency rather than durability.

Comparison with available literature data indicates that pterosaurs developed teeth more rapidly than other major extinct reptilian groups examined. The ornithocheiriform specimens analyzed (including our LPP‐UFRN 3002 with 82 von Ebner lines, MCF‐PVPH‐879‐2 with 54 lines, and *Hamipterus* with 70 lines) show formation times of 54–82 days. This contrasts with longer development periods documented in dinosaurs (185–933 lines), most crocodyliforms (50–283 lines), and mosasaurs (342–426 lines). The thin enamel in LPP‐UFRN 3002 is consistent with this rapid development pattern, as thicker enamel layers typically require extended formation times. These findings suggest that rapid tooth formation, coupled with thin enamel in ornithocheiriform pterosaurs, may have been an adaptation for reducing skull weight to facilitate flight, while maintaining tooth functionality through frequent replacement cycles.

The specimen preserves detailed microstructural features despite diagenetic alteration typical of the Crato Formation, including calcite permineralization along fractures and partial recrystallization of dentin. This preservation allows examination of growth increments and tissue organization that would otherwise be lost in more severely altered fossils.

The pterosaur tooth LPP‐UFRN 3002 demonstrates characteristic diagenetic patterns for the Crato Formation, including calcite permineralization along fractures and partial recrystallization of dentin tissue. Despite these alterations, the specimen preserves critical microstructural details, including von Ebner incremental lines, enamel layer thickness, and dentin tubule density. This preservation pattern reflects the carbonate‐saturated pore fluids typical of the Crato Formation lacustrine environment, which facilitated early cementation while introducing selective microstructural damage through calcite replacement. The contrast between the tooth's diagenetic alteration and the exceptional soft‐tissue preservation documented in other Crato Formation fossils highlights the differential taphonomic pathways affecting mineralized versus organic tissues within this Konservat‐Lagerstätte. These findings contribute to understanding the preservational limits and opportunities for histological analysis in carbonate‐dominated fossil deposits.

Future study should focus on expanding the histological database for pterosaur teeth, particularly from different clades and feeding ecologies. Additional comparative studies incorporating more crocodyliform and dinosaur taxa would help refine our understanding of tooth development patterns across archosaurs.

## AUTHOR CONTRIBUTIONS

**Tito Aureliano:** Conceptualization; investigation; funding acquisition; writing – original draft; methodology; validation; visualization; writing – review and editing; software; project administration; formal analysis; data curation; supervision; resources. **Matheus P. S. Rocha:** Writing – original draft; investigation; methodology; validation; writing – review and editing; software. **Francisco P. Lima‐Filho:** Validation; data curation; supervision; resources; writing – review and editing. **Aline M. Ghilardi:** Writing – review and editing; methodology; data curation; supervision; resources; funding acquisition; validation.

## Supporting information


**TABLE S1.** Number of von Ebner lines (vEl) and the mean thickness of von Ebner lines in pterosaurs, dinosaurs, crocodyliforms, and mosasaurs (data from the literature).

## Data Availability

The thin section is housed in a research institution and can be accessed through a request to the collection's curator.
